# Preclinical multi-target strategies for myocardial ischemia-reperfusion injury

**DOI:** 10.3389/fcvm.2022.967115

**Published:** 2022-08-22

**Authors:** Yuqing Li, Yi Gao, Guangping Li

**Affiliations:** Tianjin Key Laboratory of Logic-Molecular Function of Cardiovascular Disease, Department of Cardiology, Tianjin Institute of Cardiology, The Second Hospital of Tianjin Medical University, Tianjin, China

**Keywords:** myocardial ischemia-reperfusion injury (IRI), multi-target strategies, cardioprotection, acute myocardial infarction, apoptosis

## Abstract

Despite promising breakthroughs in diagnosing and treating acute coronary syndromes, cardiovascular disease’s high global mortality rate remains indisputable. Nearly half of these patients died of ischemic heart disease. Primary percutaneous coronary intervention (PCI) and coronary artery bypass grafting can rapidly restore interrupted blood flow and become the most effective method for salvaging viable myocardium. However, restoring blood flow could increase the risk of other complications and myocardial cell death attributed to myocardial ischemia-reperfusion injury (IRI). How to reduce the damage of blood reperfusion to ischemic myocardium has become an urgent problem to be solved. In preclinical experiments, many treatments have substantial cardioprotective effects against myocardial IRI. However, the transition from these cardioprotective therapies to clinically beneficial therapies for patients with acute myocardial infarction remains elusive. The reasons for the failure of the clinical translation may be multi-faceted, and three points are summarized here: (1) Our understanding of the complex pathophysiological mechanisms of myocardial IRI is far from enough, and the classification of specific therapeutic targets is not rigorous, and not clear enough; (2) Most of the clinical patients have comorbidities, and single cardioprotective strategies including ischemia regulation strategies cannot exert their due cardioprotective effects under conditions of hyperglycemia, hypertension, hyperlipidemia, and aging; (3) Most preclinical experimental results are based on adult, healthy animal models. However, most clinical patients had comorbidities and received multiple drug treatments before reperfusion therapy. In 2019, COST Action proposed a multi-target drug combination initiative for prospective myocardial IRI; the optimal cardioprotective strategy may be a combination of additive or synergistic multi-target therapy, which we support. By establishing more reasonable preclinical models, screening multi-target drug combinations more in line with clinical practice will benefit the translation of clinical treatment strategies.

## Introduction

Translating single, mechanistic basic experimental research to clinically beneficial outcomes is not a parallel, idealized process. Predicting clinically meaningful outcome endpoints is difficult, even from a well-recognized, robust preclinical indicator. The contradiction between successful animal research and ineffective translation of clinical effects has become an urgent problem to be solved (1). Due to improved tertiary prevention strategies, mortality in ST-segment elevation myocardial infarction (MI) patients has declined over the past 15–20 years, while the number of patients with post-MI heart failure is increasing (2). The unpredictable onset of acute MI severely limits pharmacologically protective preconditioning (1, 3, 4). However, this does not mean that cardioprotection is no longer necessary. It is just that it is increasingly difficult to demonstrate its utility, so the target population must be carefully selected. There is still, and always will be, a subset of patients who develop heart failure, especially those with the anterior wall or multiple recurrent MI. This may be why cardioprotective research is still ongoing for 50 years (5). In 2019, the COST ACTION cardioprotection consortium proposed a multi-target treatment strategy, that is, in the case of the coexistence of clinically uncontrollable variables, the rational combination of two or more different protection strategies may help produce a solid and robust cardioprotective effect (3). In this regard, we should start from the following aspects in the future to improve the possibility of successful clinical translation: (1) to deeply explore the complex pathophysiological mechanism of myocardial ischemia-reperfusion injury (IRI) and focus on the changes of the pathophysiological mechanism under the condition of comorbidities (3, 6, 7); (2) clarify the classification of specific therapeutic targets and screen out more reasonable and promising multi-target therapeutic strategies based on this; and (3) establish more reliable preclinical evaluation standards and preclinical animal models that conform to clinical practice, including animal models with multiple comorbidities and animal models receiving “background drug treatment.” In addition, the multi-target therapeutic strategies that have been screened should be able to be reproduced.

## Cardioprotective targets for myocardial ischemia-reperfusion injury: A cornerstone for therapeutic strategy development

**FIGURE 1:**
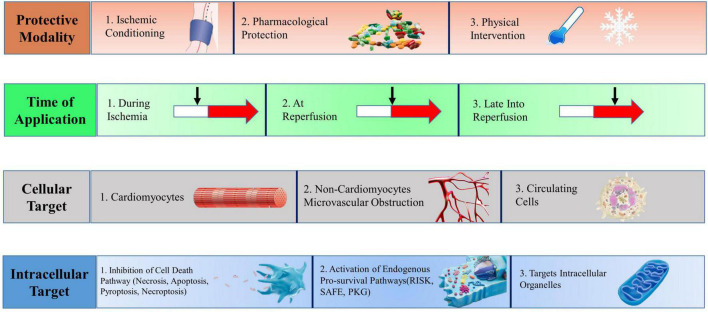
Multitarget cardioprotective strategies to reduce myocardial infarction. Cardioprotective strategies fall into four broad categories, which may be combined in different manners to achieve multitarget cardioprotection. RISK, reperfusion injury salvage kinase; SAFE, survivor activating factor enhancement; PKG, protein kinase G. [Adapted from Davidson el al. (3)].

In 1960, Jennings and colleagues (8) proposed the concept of myocardial IR based on heart studies in ischemic dogs, confirming that blood flow reperfusion leads to increased infarct size and cardiomyocyte death. In recent years, cardioprotective strategies for myocardial IRI have been continuously proposed. The classification depends on the mode of action, duration of action, cellular target, and intracellular target (Figure 1).

### Cardioprotective targets for myocardial ischemia-reperfusion injury: Classification by mode of action

Cardioprotective strategies are classified according to the mode of action, that is, ischemic conditioning (episodes of brief ischemia and reperfusion), pharmacological protection (the administration of chemical substances), and physical methods (hyperoxia, hypothermia, and electrical nerve stimulation).

The components of ischemic conditioning include ischemic preconditioning (IPC), ischemic postconditioning (IPostC), and remote ischemic preconditioning (RIC) (2). IPC reduces myocardial damage by briefly blocking blood flow for several cycles and restoring perfusion before reperfusion of ischemic myocardium. Its clinical application is limited due to the inability to determine the ischemic time of clinical patients (9). IPostC is to reduce MI size through four cycles of blood flow occlusion/reflow within a short period (usually within 1 min) after the start of blood reperfusion (10, 11). Similarly, RIC reduces MI size by inducing transient IR in other non-cardiac organs before persistent coronary occlusion. Inflation/deflation of the upper arm or thigh through the blood pressure cuff can simulate the RIC model and is widely used in clinical experiments (12, 13). RIC is uniquely attractive because it acts to stimulate away from the heart. In short, RIC produces transferable cardioprotective factors that affect downstream signaling pathways shared by the three through idiographic neurohumoral courses (14, 15). For example, extracellular vesicles accumulated in damaged myocardium during IR promote cell survival, and the underlying mechanism involves the regulation of extracellular vesicle miRNAs (16). Likewise, RIC cannot exert its cardioprotective effect under the condition of brainstem vagotomy and vagotomy of the innervating heart (17, 18). The exact interplay between neuronal and humoral pathways underlying RIC remains to be elucidated. The downstream signaling pathways shared by the three have been confirmed to include the Reperfusion Injury Rescue Kinase (RISK) pathway (PI3K/Akt and Erk1/2), the Survivor Activator Factor Enhancement (SAFE) pathway (TNF and JAK/STAT), and cGMP-protein Kinase G (PKG) pathway (10). These salvage pathways activate downstream mediators and exert cardioprotective effects by inhibiting pathophysiological processes such as calcium overload, oxidative stress, inflammatory cascades, and mitochondrial permeability transition pore (MPTP) opening (19–21).

The discovery of specific therapeutic targets in basic research provides a theoretical basis for drug research, and the cardioprotective properties of new drugs offer more therapeutic methods for clinical application (22).

Acute myocardial IRI can lead to autonomic dysfunction and is positively associated with mortality in patients with MI, making vagus nerve stimulation a promising therapeutic target (23, 24). The protective effect of hypothermia-induced IR brain injury has stimulated interest in applying therapeutic hypothermia to myocardial IRI (25). However, most physical therapy focuses on preclinical experiments, and more ideal primary research results are needed to give people confidence before entering clinical investigations (26, 27).

### Cardioprotective targets for myocardial ischemia-reperfusion injury: Classification by the duration of action

Cardioprotective strategies are classified according to the duration of action, including before ischemia, during ischemia and reperfusion, and after reperfusion. In a clinical setting, ischemia time is difficult to predict, and the myocardium is ischemia before symptoms appear, so we only discuss cardioprotective strategies after ischemia. Interventions [such as metoprolol (28–30), metformin (31), and NO donors (32)] can be cardioprotective during acute ischemia. Early administration of metoprolol or RIC may have potential cardioprotective potential during patient transport to the cardiac catheterization laboratory. However, in patients with ST-segment elevation MI, reperfusion of the ischemic myocardium with emergency PCI remains the best treatment and should not be delayed (3).

### Cardioprotective targets for myocardial ischemia-reperfusion injury: Classification by ultimate protection of intracellular target

Cardioprotective strategies can be classified according to their protective mechanisms. The first category is intracellular pro-survival signaling targets, including NO/PKG signaling cascades, SAFE, and RISK pathways (33). The second category is the regulation of cell death pathways, with targets including reactive oxygen species, MPTP, protein kinases, ion exchange channels, and inflammatory mediators. Necrosis, autophagy, and apoptosis are the three main types of cell death. Together, necrosis, pyroptosis, ferroptosis, parthanatos, and CypDmediated necrosis constitute regulated necrosis, providing theoretical support for necrotic cell regulation after MI (34). The third category targets intracellular organelles, including mitochondria (35), endoplasmic reticulum, lysosome, and nucleus. Such techniques are usually proposed based on existing pharmacological theories to solve the problem that drugs cannot act on target targets smoothly. However, these techniques rarely develop into clinical trials (36, 37).

### Cardioprotective targets for myocardial IRI: Classification by ultimate protection of cellular target

Finally, cardioprotective strategies may protect cardiomyocytes or non-cardiomyocytes such as leukocytes, monocytes, macrophages, platelets, etc. Although cardiomyocytes are most susceptible to IRI, non-cardiomyocytes, including smooth muscle cells, nerve cells, endothelial cells, and fibroblasts, are also greatly affected. Other components, including extracellular vesicles, cytokines, chemokines, etc., play signal transduction functions during IRI (38, 39). Existing experiments have demonstrated that platelets can carry and release various factors and then activate the SAFE and RISK pathways to play a cardioprotective role by mediating cardiomyocyte secreted factors (such as sphingosine-1 phosphate, stromal cell-derived factor 1α, transforming growth factor β1, and microRNAs) (40). In contrast, in addition to promoting arterial thrombosis, activated platelets can mediate the formation of microvascular microthrombi, leukocyte-platelet interactions, release vasoconstrictor molecules and microbubbles, and increase the risk of cardiac IRI through intravascular effects (41, 42). Therefore, an in-depth understanding of the protective factors released by activated platelets may contribute to developing new cardioprotective drugs, which can be combined with existing P2Y12 receptor antagonists to exert additional cardioprotective effects (43). Available evidence suggests that erythrocyte arginase tightly controls the biological activity of NO exported and exerts a significant protective effect during IRI (44). Myocardial IR can also lead to coronary circulation damage (45–47), including endothelial cell damage, erythrocyte stasis, microembolization of debris, and release of soluble factors (48), ultimately leading to microvascular occlusion and no-reflow and intramyocardial hemorrhage. Stabilizing endothelial cells and protecting pericytes may be potential targets for preventing IRI (41, 49).

## Therapeutic strategy for myocardial ischemia-reperfusion injury: Choosing the appropriate target population and time point

### Treatment strategies should be applied to appropriate populations

The etiology of the acute coronary syndrome is mainly the rupture of cholesterol plaques caused by inflammation-induced platelet-rich thrombus formation. Other etiologies include plaque erosion, coronary spasm and embolism, calcified nodules, and spontaneous coronary dissection (50). The size of MI is jointly determined by ischemia and reperfusion-induced injury, and prognostic factors include (1) the size of the ischemic area at risk; (2) the duration and continuity of coronary occlusion; and (3) the blood supply of residual collateral circulation and the degree of microvascular dysfunction (46). The prevention of microvascular occlusion may reduce the incidence of adverse cardiovascular events after IR more than limiting infarct size (3, 41, 45). Experiments have shown that myocardial hemorrhage drives MI dilation after reperfusion and impairs myocardial salvage (19, 20, 51). Myocardial ischemia may act as one of the determinants of MI size and play an independent clinical predictive role (52). Future clinical trials must focus on patients who genuinely need adjunctive cardioprotection, i.e., severe hemodynamic changes.

### The treatment strategy should choose an appropriate time point

**FIGURE 2:**
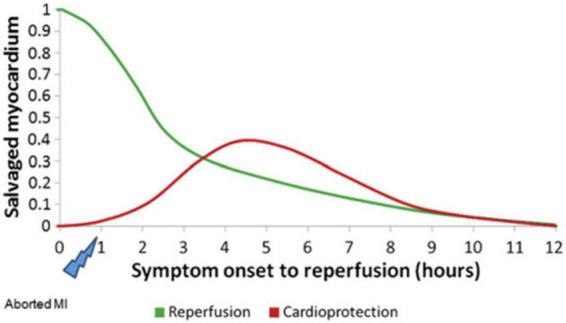
Hypothetical temporal relationship between reperfusion and cardioprotection in STEMI [Adapted from Bainey and Armstrong (27)].

Since most cell death occurs within the first few minutes of reperfusion, therapeutic strategies should be used early after IR. Loss of cardioprotective effects of drugs (such as insulin) was observed if treatment was extended to 15 min (33, 53). Myocardial protection is also temporal: early reperfusion may reduce or even terminate myocardial necrosis and prevent reperfusion injury. However, with reperfusion of the myocardium at 2–6 h, the effect of salvaging the myocardial area dropped dramatically. After 12 h, the reperfused myocardium can no longer recover (27) (Figure 2). The time curve of myocardial protection indicates the strong protective ability of early reperfusion, and exogenous myocardial protection measures are challenging to produce the effect. Exogenous interventions can be protective when reperfusion is delayed but cardiomyocytes are still viable. Future clinical trials of novel cardioprotective agents should demonstrate drug susceptibility in specific populations at different times of myocardial ischemia to obtain a potential “sweet spot” (54, 55).

### The pathophysiological mechanism of myocardial ischemia-reperfusion injury should be further explored

Reperfusion as a method to rescue ischemic myocardium has been controversial since it was proposed (21, 56–58). With an in-depth understanding of the pathophysiological mechanism of IRI, coupled with the breakthrough results of cardioprotective strategies in preclinical experiments, can people maintain confidence (59, 60)?

## The pathophysiological mechanism of myocardial ischemia-reperfusion injury

**FIGURE 3:**
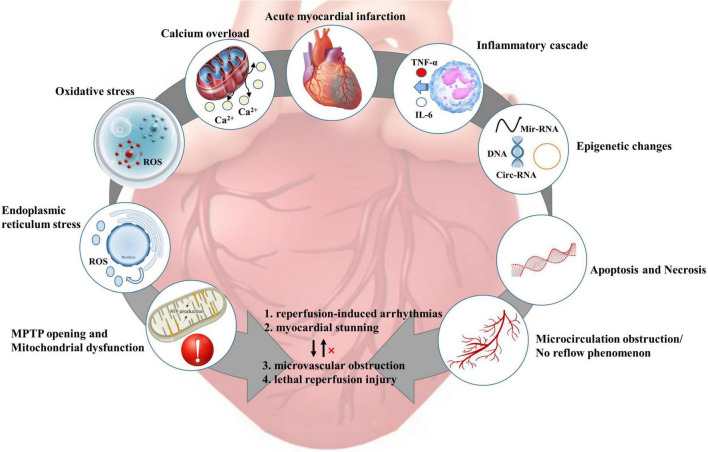
Pathophysiological mechanism of myocardial ischemia-reperfusion injury [adapted from Heusch et al. (4)]. ROS, reactive oxygen species; MPTP, mitochondrial permeability transition pore; TNF-α, tumor necrosis factor-α;IL-6, interleukin-6.

To better explain the inconsistency between the positive results of animal experiments and the problematic translation of clinical practice, it is clinically relevant to discuss the pathophysiological mechanisms of myocardial IRI. Figure 3 shows the appropriate pathophysiological mechanisms during myocardial IRI.

### Calcium overload, oxidative stress, and mitochondrial dysfunction

After coronary occlusion, anaerobic glycolysis is enhanced in the ischemic myocardium, and H^+^ accumulation leads to a decrease in intracellular pH and an increase in Na^+^/H^+^ exchange. Abnormal inactivation of 3Na^+^/2K^+^-ATPase due to decreased ATP production. Intracellular Na^+^ overload leads to abnormal activation of the 2Na^+^/Ca^2+^ exchanger, leading to calcium overload. Toxic substances such as oxygen free radicals generated during IR can damage the cell membrane structure, leading to an imbalance of intracellular calcium storage-release (61–63). Excessive intracellular calcium entering mitochondria leads to mitochondrial calcium overload, resulting in disturbance of mitochondrial energy metabolism and ultimately induction of cardiomyocyte apoptosis (37, 64, 65).

### Endoplasmic reticulum stress, epigenetic changes, and the inflammatory cascade

Endoplasmic reticulum stress (ERS) activates the unfolded protein response to degrade and clear abnormal proteins during IRI. Dual protective/degradative effects of ERS during revascularization make it impossible to demonstrate the protective effect of ERS on ischemic myocardium. Likewise, drugs related to ERS in clinical practice remain to be developed (66). Epigenetic changes, including non-coding RNA, DNA, and histone modifications, are emerging therapeutic targets because of their close relationship with the pathogenesis of IRI (67). There are comments that IRI is driven by inflammation through the interaction of multiple pathways, which mediates cardiomyocyte death. There are also comments that inflammation is merely an adaptive response to infarction (68).

### Apoptosis and necrosis

To date, six distinct forms of regulatory cell death have been observed in cardiac pathology: apoptosis, necroptosis, mitochondrial-mediated necrosis, pyroptosis, ferroptosis, and autophagic cell death [for details, please refer to Ref. (69)]. Necrosis and apoptosis are two separate cell death processes: (1) Apoptosis, an apoptotic body-mediated programmed process involving professional phagocytes. (2) Necrosis is an unregulated, passive cell death process that ultimately triggers an inflammatory cascade leading to cell swelling and loss of integrity. Recent studies have speculated that apoptosis and necrosis may be interconnected during myocardial IRI or continuously occur. However, the respective contributions of apoptosis and necrosis in cardiomyocyte death remain unclear (70, 71). There are comments that massive necrosis of cells during myocardial ischemia and activation of pro-apoptotic pathways after reperfusion induces aggravation of cardiomyocyte apoptosis (72). Most cardiomyocytes die within 24 hours of coronary occlusion. Pro-inflammatory responses and biological stress in the infarct zone may trigger a second wave of cardiomyocyte death, but its intensity is significantly reduced (4, 73).

### Microcirculation obstruction/no reflow phenomenon

Microcirculatory perfusion cannot be restored after recanalizing ischemic coronary vessels, mainly manifested as prominent low perfusion area and severe inhibition of blood flow (4, 74, 75). No-reflow is the most severe form of myocardial IRI in microcirculation. No-reflow is as essential a poor prognostic factor in clinical patients as intramyocardial hemorrhage. Epicardial coronary blood flow can be restored in clinical PCI procedures, but distal coronary perfusion is incompletely restored in about half of patients. Pre-PCI comorbidities, such as diabetes, hypertension, hypercholesterolemia, and smoking, may increase the risk of microcirculatory reperfusion injury in clinical patients (41, 76, 77).

## Multi-target strategies for myocardial ischemia-reperfusion injury: Application in animals with co-morbidities

**FIGURE 4:**
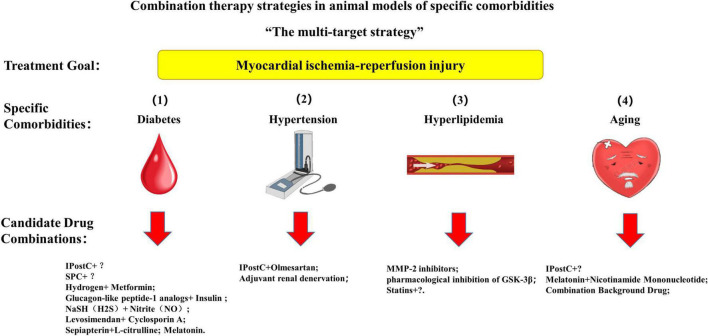
A multi-target strategy for myocardial ischemia-reperfusion injury in animal models with specific comorbidities. IPostC, ischemic postconditioning; SPC, sevoflurane postconditioning.

Multitarget cardioprotective therapy is defined as multiple cardioprotective agents or interventions targeting different targets acting together to exert additive or synergistic cardioprotective effects. A single intervention strategy can also be considered a multi-target strategy if it protects against multiple targets (3). To solve the contradiction between the significant cardioprotective effect obtained by a single cardioprotective strategy in animal experiments and the failure of clinical translation is the original intention of the multi-target strategy. One of the common denominators of the shortcomings of multiple translational trials is that patients have other specific comorbidities in addition to the application of PCI (1, 3, 22, 45, 59, 71, 78–80). Consequently, we hypothesize that the multi-target strategy for protecting myocardial IRI in the animal testing stage may be applied to specific co-morbidities, exert a more substantial cardioprotective effect, and better guide clinical trials (81). We summarize preclinical multi-target cardioprotective strategies for myocardial IRI and other comorbidities, including diabetes, hypertension, hyperlipidemia, and aging (Figure 4).

### Myocardial ischemia-reperfusion injury combined with diabetes mellitus

Diabetic patients have increased adverse cardiovascular events and worse clinical outcomes after acute MI. Despite the lack of clinical evidence, animal studies suggest diabetic hearts are resistant to cardioprotective strategies (82). A review concludes that hyperglycemia initially causes the heart to become resistant to IR, contradicting our understanding. However, its mechanism may be related to activating the Akt/hexokinase II pathway due to the increase of insulin levels in the body under hyperglycemia. IRI is exacerbated when diabetes and insulin resistance develops (83).

#### Hyperglycemia inhibits the cardioprotective effects of sevoflurane preconditioning and ischemic regulation strategies

Diabetes increases myocardial susceptibility to IRI and alters the myocardial response to ischemic regulatory strategies by disrupting normal intracellular signaling (84). Diabetes-induced blockade of cardioprotective is caused by the diseased myocardium, independent of humoral factors released by RIC. The research should focus more on blocked signaling cascades in diseased myocardium under diabetic conditions (85). Maintaining or enhancing normal intracellular protective signaling, thereby reversing the cardioprotection of hyperglycemia-suppressing ischemic regulatory strategies, becomes the therapeutic target of multi-target systems. In cardiovascular risk factors such as diabetes, autophagy’s protective and harmful effects on myocardial IRI must be clarified further (86).

Myocardial ischemia from cardiac surgery may also lead to IRI. Brief administration of a volatile anesthetic before initiation of reperfusion, known as anesthetic postconditioning, reduces MI size and improves cardiac function. The study confirms that sevoflurane postconditioning (SPC) protects normal rat hearts from IRI, and its mechanism depends on NO-mediated recovery of autophagic flux (87). However, SPC has no protective effect on diabetic myocardial IRI, confirmed in a diabetic mice model of myocardial IRI. The reason may be related to the impaired activation of the PTEN/PI3K/Akt signaling pathway mediated by TOPK (88).

#### A multi-target strategy based on ischemic regulation under hyperglycemia

Increased expression of autophagy activation-related proteins in isolated rat hearts exerts cardioprotection. However, no increase in autophagy activation-related protein expression was found in left ventricular biopsies from CABG patients undergoing IPC (89). This discrepancy between preclinical and clinical experimental results makes the cardioprotective function of autophagy more controversial. Studies have shown that the combined effect of IPostC and alpha-lipoic acid in diabetic rats’ hearts can reduce the infarct size of isolated diabetic hearts by restoring autophagic flux and mitochondrial function. The experimental results coincide with the common expectation that induction of controlled autophagy at the appropriate time and level and inhibition of inappropriate autophagy can exert cardioprotective effects (90). However, the experiment did not give the specific protective mechanism of the cardioprotective strategy alone. Whether or not the autophagy-induced cardioprotective mechanism is jointly activated needs to be verified experimentally (91). Strong experimental evidence indicates that the cardioprotective effect induced by IPostC is related to the up-regulation of PI3K/Akt survival signaling pathway and MPTP inhibition, and the specific mechanism involves the inactivation of GSK-3β and the increased expression of anti-apoptotic proteins such as Bcl-2. However, the cardioprotective effect of IPostC is counteracted by the hyperresponsiveness to IR in diabetic patients (92). Co-administration of additional drugs that protect PI3K/Akt/GSK-3β signaling [such as thymoquinone (93) and cyclosporine A (94)] reversed the inhibitory effect of chronic hyperglycemia on IPostC and provided additional cardioprotection in diabetic rats’ hearts. Therefore, we can reasonably assume that the application of drugs that protect other signaling cascades, including SAFE and NO/PKG signaling cascade, combined with IPostC, can exert the same additional cardioprotective effect, which needs further experimental verification.

#### A multi-target strategy based on SPC under hyperglycemic conditions

Zhang et al. (95) confirmed that hydrogen sulfide (H_2_S) attenuates hyperglycemia-induced oxidative stress and mitochondrial dysfunction induced during myocardial IR in hyperglycemic rats by enhancing the SIRT1/NrF2 signaling pathway and restores the cardioprotective effect of SPC. This experiment is the first to explore the relative roles of STAT1\/Nrf2 signaling in regulating mitochondrial dysfunction and oxidative stress in H2S, restoring SPC-mediated cardioprotection in diabetic rats. Diabetes also leads to impairment of the JAK-STAT3 pathway. The antioxidant N-acetylcysteine restores the cardioprotective results of SPC in diabetic rats by enhancing STAT3 activity and adiponectin and reducing Fox1 and CD36 (96). This experiment also prospectively expresses expectations for the combined therapeutic effect of N-acetylcysteine and IPostC. Deferoxamine acts as an iron chelator to stabilize HIF-1α expression, improve HIF-1α activity in hyperglycemic states, and protect diabetic rats’ hearts. Deferoxamine restores HIF-1/BNIP3-mediated mitophagy, reduces ROS production, and restores mitochondrial function, ultimately restoring the protective effect of SPC on diabetic rats’ hearts (97, 98).

#### A multi-target strategy based on hypoglycemic drugs under hyperglycemic conditions

Studies have shown that metformin is more effective than sulfonamides and insulin in reducing all-cause mortality and diabetes-related end events. However, gastrointestinal reactions, drug-induced dermatitis, and lactic acidosis became apparent after a period of clinical use in patients. The liver and kidney damage of metformin limits the use of high doses of metformin. In this regard, metformin-based combination drug therapy has been widely concerned. The study found that the combined impact of hydrogen and metformin on the protection of diabetic mice myocardium is better than that of metformin monotherapy (99). Glucagon-like peptide-1 analogs combined with insulin therapy can significantly affect myocardial IRI in diabetic rats (100). Vildagliptin has also been shown to restore the protective effect of IPostC on diabetic rats’ hearts (101). However, the experimental study did not give the specific mechanism of the cardioprotective influence of hypoglycemic drugs but only a particular elaboration of the observed infarct size phenomenon. Other combination therapy strategies have explored the specific means of glucose-lowering medications. Still, they have not been tested in animal models of diabetes, thus circumventing the effects of hyperglycemic conditions on drug interactions (102).

#### Other multi-target strategies in hyperglycemic conditions

Studies have shown that low-dose NaSH alone does not exert cardioprotective effects. NaSH combined with nitrite increases the cardioprotective effect of nitrite in type 2 diabetic rats by enhancing cystathionine γ-lyase and endothelial NOS expression (103). Experiments suggest that the combination of H2S and NO may provide more therapeutic value and explore more potential drug combinations. The cardioprotective effect of post-treatment with the Ca^2+^ sensitizer levosimendan was inhibited under hyperglycemia. Different experimental settings *in vivo* and *in vitro* resulted in the opposite results on whether the increase of levosimendan concentration could have a protective effect on the diabetic rats’ hearts. However, it is impractical to use levosimendan at 10-fold higher concentrations (104, 105). Cyclosporine A, an MPTP opening inhibitor, reverses the loss of cardioprotective effects of levosimendan in diabetic rats under high glucose environments, suggesting the possibility that increased drug stimulation can change the loss of cardioprotective function. Diabetic heart disease increases tetrahydrobiopterin oxidation and increases arginase activity, uncoupling NO production. Sepiapterin acts as a stable precursor of tetrahydrobiopterin, and L-citrulline is an efficient precursor of L-arginine. The combined action of the two overcomes the protection limitation of a single drug. It protects coronary endothelial function and nitric oxide production by protecting the dimerization of endothelial NO synthase and exerting the effect of resisting diabetic mice myocardial IRI (106). As a targeted mitochondrial quality control drug, Melatonin attenuated the progression of diabetic cardiomyopathy and reduced the vulnerability of diabetic rats’ myocardium to IRI by mediating the SIRT6-AMPK-PGC-1α-AKT axis to maintain mitochondrial quality control (107).

### Myocardial ischemia-reperfusion injury combined with hypertension

#### Hypertension inhibits the cardioprotective effects of ischemic regulation strategies

Studies have found that pretreatment with ischemic regulation strategies and pharmacological strategies (e.g., adenosine receptor agonists, propofol) can reduce MI size in normal rats (108, 109). However, hypertensive rats treated with IPostC did not achieve a reduction in infarct size. Likewise, IPostC increased GSK-3β phosphorylation in normotensive rats, but this increase was not present in hypertensive rats (110). Cardiac hypertrophy also increases myocardium susceptibility to IRI, impairing the cardioprotective effects of IPostC by inhibiting Akt phosphorylation. Ultimately IPostC failed to reduce infarct size (111).

#### A multi-target strategy based on ischemic regulation under hypertension

An effective cardioprotective strategy is to reduce angiotensin II production (using angiotensin-converting enzyme inhibitors or angiotensin receptor blockers) (112, 113). However, there are still few studies on hypertrophic cardioprotective preconditioning (114, 115). Studies have confirmed that long-term olmesartan treatment can alleviate left ventricular hypertrophy by downregulating the expression of HIF-1a, miR-21, and miR-210 and finally restore the cardioprotective effect of IPostC in spontaneously hypertensive rats (116). Chronic captopril treatment reduces ventricular hypertrophy and infarct size in spontaneously hypertensive hearts. Confusingly, acute captopril combined with IPostC is not protective in hypertensive hearts. The reason does not exclude that the antioxidant effect of captopril can avoid the cardioprotective effect of IPostC (110).

#### Other multi-target strategies in hypertension conditions

Renal denervation therapy, a novel therapeutic modality for refractory hypotension, has been proven to protect hypertensive rat myocardium from IRI by improving NO bioavailability, inhibiting oxidative stress and GRK2 signaling (117). Renal denervation reduces myocardial fibrosis and improves left ventricular function in patients with heart failure, which relies on increasing circulating natriuretic peptide levels (118). Early cardiosphere-derived cells combined with adjuvant renal denervation improve left ventricular ejection fraction and ventricular remodeling, suggesting a novel combined alternative to cell therapy after MI (119). Recent studies have found that renal denervation therapy inhibits the outflow of myeloid cells from the spleen and protects myocardial ischemia mice from reperfusion injury by protecting splenic immune cell mobilization (120). This experiment revealed a new link between sympathetic nerve activity and the inflammatory response to myocardial IRI. Whether combined anti-inflammatory intervention and renal denervation therapy can exert additional cardioprotective effects needs further investigation.

### Myocardial ischemia-reperfusion injury combined with hyperlipidemia

Studies found that hyperlipidemia interferes with cardioprotective signaling and directly damages the myocardium (80, 115). Hyperlipidemia leads to increased oxidative stress, mitochondrial dysfunction, and inflammation-induced apoptosis during myocardial IRI, which may account for myocardial dysfunction and increased susceptibility of the myocardium to infarction (121, 122). Hyperlipidemia needs to be considered one of the comorbidities that interfere with cardioprotection.

#### Hyperlipidemia inhibits the cardioprotective effects of ischemic regulation strategies

Ischemic regulatory strategies have a strong protective effect on myocardial IRI. However, the cardioprotective effects of IPC, IPostC, RIC, and SPC were inhibited in hyperlipidemia (123–126). Osipov et al. demonstrated enlarged MI size in a porcine model of hyperlipidemia (122). Likewise, cardioprotective effects of ischemic modulation strategies have been shown to be lost in different hyperlipidemia rat models (127). In one study, however, IPostC was still therapeutically effective in hyperlipidemic rats. The underlying mechanism may be that hyperlipidemia does not affect the up-regulation of HIF-1α, which provides a new therapeutic idea for treating hyperlipidemia (128).

#### NO-cGMP-PKG signaling pathway fails to exert cardioprotective effect under hyperlipidemia

Once considered a promising therapeutic target, the NO-cGMP-PKG signaling pathway is one of the most extensively studied protective pathways against hyperlipidemia. However, NO donor pretreatment did not significantly reduce MI size in hypercholesterolemic animals, and the mechanism may be related to the inactivation of PKG oxidative dimerization (32, 80, 115, 123). The cardioprotective effect of the ATP-sensitive K^+^ channel activator diazoxide is also inhibited under hyperlipidemic conditions (129). Experiments targeting the MPTP have yielded conflicting results. On the one hand, neither IPostC nor cyclosporine A could exert cardioprotective effects in hyperlipidemia rats (130). On the other hand, experiments confirmed that combining the two could restore the cardioprotective development of IPostC (131). However, neither group of experiments made a specific signaling pathway explanation for the results, and the reason for the positive results was only based on observing simple phenomena.

#### Other promising therapeutic targets for cardioprotection in hyperlipidemic conditions

Experiments suggest that a matrix metalloproteinase-2 inhibitor (MMP-2) plays a protective role in hyperlipidemic hearts (132). The cardioprotective effects of MMP-2 inhibitors are independent of RISK/mPTP signaling, paralleling known cardioprotective pathways (133). RISK pathways also play a cardioprotective role in hyperlipidemia. Studies have shown that the PTEN inhibitor bisperoxovanadium can restore the cardioprotective effect of RIC in hyperlipidemia rats, which may be related to promoting downstream Akt/GSK-3β phosphorylation (134). In hyperlipidemia research, pharmacological inhibition of GSK-3β may be a promising future therapeutic target (135). The cardioprotective effects of statins are independent of their lipid-lowering products. Cardioprotective effects of statins are blocked in an animal model of hyperlipidemia as in IPostC (136). Combination therapy with statins and other cardioprotective strategies becomes a potential multi-target strategy for addressing hyperlipidemia with myocardial IRI.

### Myocardial ischemia-reperfusion injury combined with aging

#### Aging inhibits the cardioprotective effects of ischemic regulation strategies

Aging can block the protective effects of ischemic regulation strategies against myocardial IRI (80, 115, 137). A study using endothelial function as an endpoint found that increasing age was associated with the loss of IPC on the protective function of the brachial artery endothelium (138). Similarly, many studies have focused on changes in the components of relevant signaling cascades with aging, evaluating differences in adult versus old cardiac function, which may help explain the observed loss of cardioprotection with age (139).

#### A multi-target strategy based on ischemic regulation under aging

Studies have confirmed that exogenous H_2_S can upregulate autophagy by activating the AMPK/mTOR pathway in aging hearts, thereby restoring the cardioprotective effect of IPostC (140). Nicotinamide mononucleotide preconditioning in aging hearts restores the cardioprotective results of IPostC by protecting mitochondrial function (141). Interestingly, applying the tandem effect of training combined with food restriction can restore the protective effect of IPostC against IRI in aging hearts by restoring norepinephrine release (142). However, these studies have focused on IRI in isolated hearts *in vitro*, and it is still inconclusive whether the *in vivo* experiments can exert the same effect. Potential drug combinations still need to be tested in large animals to obtain clinical translational evidence support.

#### A multi-target strategy based on mitochondria protection under aging

Increased production of ROS in aging cardiomyocytes leads to mitochondrial dysfunction, leading to increased susceptibility of aging hearts to ischemia (143). A study on aged rats confirmed that melatonin and nicotinamide mononucleotide combined effect could alleviate myocardial IRI by reducing mitochondrial oxidative stress and ROS generation (144). Cardiac aging may be associated with calcium-induced MPTP opening and increased susceptibility to the mitochondrial release of cytochrome C. Studies have demonstrated that melatonin inhibits both events by protecting cardiolipin from ROS damage during reperfusion preventing cardiomyocyte necrosis and apoptosis (145).

#### Other promising therapeutic targets for cardioprotection in aging conditions

Other combination strategies have not yet been tested in animal aging models but show promising promise. Hydrogen combined with metformin has been shown to have an excellent protective effect on diabetic cardiomyopathy (99). Metformin rescues autophagy defects by inhibiting p62 accumulation and protects the aging myocardium from IRI (146). Similarly, H_2_ combined with CO can show an enhanced therapeutic effect against myocardial IRI through anti-inflammatory and antioxidant mechanisms (147). Combination therapy of metformin, H_2_, and CO may vigorously protect against IRI in aging hearts. The anti-aging drug dasatinib combined with quercetin has shown an excellent protective effect in patients with idiopathic pulmonary fibrosis and diabetic nephropathy. Whether connecting the two drugs can translate into cardioprotection in the elderly requires more preclinical evidence (148).

## Clinical translation of multi-target strategies for myocardial ischemia-reperfusion injury: How can we move forward

No trials have demonstrated clinical benefit despite attenuating myocardial IRI on the bench. Thus, the hope for clinical application of cardioprotective therapy is fading. Heusch (1) calls for “Cardioprotection research must leave its comfort zone.” We are all describing bright application prospects for the cardioprotective effects obtained in preclinical research, but few people rationally consider how to translate preclinical experiments into clinical trials. Rossello and Yellon (149) call this phenomenon a “disconnected paradigm,” a complete disconnect between preclinical and clinical cardioprotection studies. They propose to add similar bench conditions to the clinical setting, i.e., “Cardioprotection needs to go backward before it can move forward.” They describe cardioprotection translation as a 4-step process, starting from (1) simplified animal studies, (2) clinically relevant animal studies, (3) clinical proof-of-concept studies with surrogate endpoints, and (4) clinical outcomes experiments (149). Here, we will discuss possible issues with current cardioprotective translation for researchers to adopt before beginning clinical studies. The aim is to improve the possibility of translating novel cardioprotective measures into clinical applications.

### We need *in vivo* standards for preclinical assessments that are sufficiently reliable to support clinical trials

Based on the gradual revealing of the pathophysiological mechanisms of myocardial IRI, mechanical and pharmacological cardioprotective strategies targeting related signaling pathways and molecular targets have been identified. Animal experiments aim to establish new mechanisms determined by their nature reductionist (150).

#### Systematic and rigorous preclinical evaluation criteria

Therefore, the IMproving Preclinical Assessment of Cardioprotective Therapies (IMPACT) criteria were proposed (81). The IMPACT criteria divide preclinical experiments into three steps. (1) Step 1: Small animal model; (2) Step 2: Small animal model (confounders); (3) Step 3: Large animal model. The IMPACT criteria focus on the same points in the different steps. The establishment of animal models should include acute ischemia and reperfusion (non-permanent occlusion to represent the clinical situation better) (20, 41). Cardioprotection’s endpoint should be the infarct size relative to the risk area (coronary microvascular occlusion is another critical endpoint) (151). The IMPACT criteria set its minimum requirements to validate infarct size and microcirculatory damage in a single-center acute IRI model (minimum 2 h, optimal at 24 h) in a single species (e.g., mouse, rat, or rabbit). Reperfusion time can be extended to 72 h in large animals such as pigs. In contrast, ideal criteria were validated infarct size and left ventricular remodeling in male and female models of chronic IRI (minimum 28 days in small animals and 3 months in large animals) (19, 46). It needs to be re-validated to make the experimental effect closer to the clinic in the presence of two or more confounding factors. Although not all steps are relevant to the evaluation of mechanical or pharmacological cardioprotective strategies, successful implementation of more steps will help reduce the risk of failure to translate novel cardioprotective measures clinically.

#### Multi-target strategy should go deep into specific mechanism research

The research on preclinical multi-target strategies focuses on discovering cardioprotective phenomena, and the specific protective mechanisms of different cardioprotective strategies cannot be determined (93, 100, 116, 131). Meanwhile, most experiments focus on isolated hearts and small animal models such as mice, rats, and rabbits. Large animal models (such as pigs) are rarely studied due to the complexity of experimental design, experimental conditions, research costs, and experimental regulatory requirements (130, 131, 141, 144, 148). Exenatide and RIC exert additive effects on cardioprotection in porcine myocardial IRI by activating distinct cardioprotective pathways, which looks very attractive for clinical translation (152). However, no short-term clinical benefit in infarct size reduction was observed with exenatide and RIC alone or combined (153). The failure of clinical trial translation also reminds experimenters that clinical trials should be supported by sufficient preclinical evidence.

### The establishment of preclinical animal models needs to be closer to clinical practice

One of the reasons for the failure of the clinical translation may be the significant difference in comorbidity status between preclinical animals and clinical patients. Therefore, the development of animal models that are closer to clinical practice has become an urgent issue.

#### Preclinical cardiomyocyte model

Developing novel cardiomyocyte culture strategies may be a potential model-building approach (154, 155). A recent experiment successfully proposed the first *in vitro* aging myocardial tissue model based on human-induced pluripotent stem cell-derived cardiomyocytes, providing a promising new platform for studying cardiovascular disease and other age-related diseases (156). This experiment is also the first to demonstrate that age-appropriate *in vitro* disease models can be developed to provide more cutting-edge physiological insights into cardiovascular disease development, progression, and improvement.

Similarly, most human specimens are used for isolated cell preparation. The overall structure and function of the cells after enzymatic digestion are greatly affected, and the lack of multicellularity makes this cell model unsuitable for studying pharmacological reactions. A recent survey produced living myocardial slices with a high-precision vibrating group, which preserves the natural multicellular structure of the heart. Its ultra-thin (100–400 μm) thickness allows nutrients to diffuse to the innermost cells, maintaining viability, and preventing ischemic damage *in vitro* without blood perfusion. Living myocardial slices overcome most limitations of other *in vitro* models, preventing significant structural and functional changes associated with chronic *in vitro* cultures (157, 158).

#### Human atrial preparation technology

Human atrial preparation technology has also received extensive attention. The study by Kleinbongard et al. (159) demonstrated that the atrial myocardium’s mitochondrial and contractile function might reflect the RIC’s cardioprotective effects. Although the experimental conclusions cannot be directly extrapolated to the left ventricular tissue due to the limitations of the atrial myocardium, the establishment of atrial preparation technology provides a new idea for exploring the causal relationship between signaling and protection. Similarly, establishing refined animal models can better reveal the specific targets of cardioprotection. By establishing a mouse model in which telomerase reverse transcriptase (TERT) is expressed only in mitochondria or the nucleus, it was found that the increase of mitochondrial TERT (non-nuclear TERT) has a protective effect on myocardial IRI in mice (160). Although this experiment did not reveal the specific details of how mitochondrial TERT affects inhibin and complex I, thereby improving mitochondrial respiration, this technology provides a new idea for clarifying the targeted protection sites of target factors.

#### Isolated perfused heart model

Transgenic mouse hearts are widely used for human heart disease research. However, the standard mouse heart’s fast beating (400–600 bpm) limits its ability to evaluate its kinetics. In a recent experiment, isolated mouse hearts were stabilized at 120–130 bpm at 37°C by applying 300 μM lidocaine (161). The mouse heart’s positive inotropic and gluco-optic responses were preserved, and the Frank-Starling response was enhanced. While inherent differences compared to normal hearts remain, it extends the usefulness of this transgenic mouse model in human heart disease research.

#### Small/large animal models

Most experiments in large mammals anatomically and physiologically closer to humans than mice and other rodents have been performed in young, healthy animals that lack the confounding factors specific to patients with clinical MI. To date, there are no reliable data from large animal models addressing confounding factors in cardioprotection (80). Similarly, small animal studies are more likely to incorporate only a single confounding factor (diabetes, hypertension, hyperlipidemia, etc.). Therefore, preclinical experiment design needs closer to clinical reality to obtain more conducive results for clinical translation (6). A recent study administered a combined background drug mimic of an opioid agonist, heparin, and a platelet inhibitor in rats before MI and found that the background drug was cardioprotective and independent of additional cardioprotective strategies (162). The results of this experiment may explain the failure of the translation of some cardioprotective strategies in the clinic; that is, patients have been treated with background drugs before the application of additional cardioprotective strategies. The successful application of the model could provide a new experimental platform to evaluate the effectiveness of novel cardioprotective strategies. A retrospective analysis of patients undergoing elective coronary bypass grafting with or without RIC before ischemic cardioplegic arrest found no impact of β-blockers, statins, ACE inhibitors, ARBs, or intraoperative nitroglycerin (163). This example, combined with clinical patients’ comorbidities and co-medication, shows that it is impossible to establish a “perfect” animal model relevant to all patients. Therefore, future translation of clinical trials should be strictly limited to recruiting patients with a view to discovering cardioprotective regimens in special populations.

### Preclinical experiments should be rigorous and reproducible

In addition to the lack of reliable preclinical data to support translational strategies for cardioprotection, issues of rigor and reproducibility of preclinical studies are more general concerns. Lack of blinding and randomization principles, small data volume, and statistical testing methods with free significance thresholds lead to high false-positive results that cannot be replicated in preclinical experiments.

#### Why preclinical experiments need rigor and reproducibility

Multicenter tests that consider the robustness of interventions to numerous confounders and unknown variables are an exception in studies of cardioprotective strategies (164, 165). The few reproducibility studies conducted in the biomedical field show that only 10–25% of small preclinical studies can be successfully reproduced. Preclinical results may be difficult to replicate due to biological complexity and heterogeneity barriers. Many studies also suffer from design bias, insufficient statistical power, or lack of adherence to reporting standards. These problems can lead to wasted time and resources, reduced funding, and even halting potential treatment strategies, but these problems are entirely avoidable (151, 166). In order to prove that preclinical experiments have practical significance for guiding clinical transformation, it is necessary to establish an organized multi-center network and testing standards for rigorous testing of preclinical data.

#### Our efforts to facilitate more successful clinical translation of preclinical experiments

To this end, the National Heart, Lung, and Blood Institute-sponsored and formed a clinical trials network as a model for developing a shared collaborative infrastructure for research, named CAESAR (Consortium for Preclinical Evaluation of Cardioprotective Therapeutics). The consortium will study promising heart-protective therapy in mouse, rabbit, and pig models of myocardial IRI in a manner similar to a multicenter clinical trial (in a multicenter, blinded, randomized, independent core data, and statistical analysis). The overall goal of CAESAR is to screen for truly effective cardioprotective strategies in limiting infarct size through rigorous preclinical evaluation and to provide recommendations for clinical trials to test these cardioprotective strategies (167, 168). In less than four years of CAESAR’s existence, no cardioprotective effects were found among the three interventions (sildenafil, sodium nitrite, and chloramphenicol succinate) reported extensively. The several remaining therapies have a protective effect on the heart but do not reduce the infarct size. Sadly, CAESAR remains the only public network that has performed rigorous, multicenter testing of cardioprotective therapies proposed by external investigators. Although short-lived, its work has shown that applying clinical trial-like rigor significantly impacts the results of preclinical studies (5). Recently, the acronym for Spanish network-center for cardiovascular biomedical research has set up the “Cardioprotection Large Animal Platform” (CIBER-CLAP), which will also follow the CAESAR model to verify the protective effect of IPC in a pig model of acute MI (169). It is reassuring that the seeds CAESAR sowed have germinated and borne fruit. There will eventually be a fundamental change in current approaches to cardioprotection to incorporate these stringent criteria into preclinical studies.

## Concluding remarks

In animal models of IRI, various cardioprotective therapies can effectively reduce infarct size. However, conventional animal models of IRI cannot adequately reproduce the phenomenon of IRI in patients. Here, we envision that multi-target cardioprotection is necessary to achieve therapeutic effects in these animal models and effectively apply cardioprotection to patients. Interventions combined with drugs with robust mechanisms of action, efficacy, and safety tested in preclinical experiments, combined with appropriate routes of application, are good candidates for translational clinical trials. At the beginning of such experimental designs, a factorial method could demonstrate the additive effect of combination therapy, but this approach would increase the number of patients required. Therefore, it may be better to test the combination in patients and controls by confirming the additive effect in an animal model.

Another factor that must be considered is that most clinical patients receive drug therapy before reperfusion therapy and have different comorbidities. There are no reliable data to fully demonstrate the cardioprotective effect of a multitarget strategy in large animals with IRI presence of confounding factors. Therefore, preclinical animal models should be as close to clinical reality as possible to confirm the practical protective effect of multi-target strategies.

Animal experiments on cardioprotection need to improve rigor and reproducibility, and establishing a multi-center experimental network similar to the standard of clinical trials would be beneficial. Reliable preclinical data must be available to support tests of cardioprotective strategies in humans. The transition from single-center animal experiments to initial clinical proof-of-concept trials, and even directly to clinical outcome trials, is somewhat unreasonable and disorganized. Such an approach is undoubtedly burying heart protection strategies in the grave. In the era of personalized medicine, clinical outcome studies should be conducted in carefully selected patient cohorts to prove or deny efficacy. While specific cardiac assist interventions are protective in some patients, they are not a problem as long as they are safe for most patients. Modern oncology research can develop highly effective treatments for selected small cohorts, as can current cardiovascular medicine research.

Finally, based on the examples we discussed in previous sections, some potential combination therapies for multi-target cardioprotective strategies include:

(1) RIC combined with a drug with a different mechanism of action.
(2) A drug that inhibits the cell death pathway and a drug that activates an endogenous cardioprotective pathway.
(3) A single drug acts on different protection targets, such as cyclosporin A.
(4) Background drug combination therapy (activation of RISK pathway, SAFE pathway, or cGMP/PKG pathway).

Establishing an animal model with the coexistence of multiple confounding factors and confirming that the multi-target strategy has a cardioprotective function in this model will have guiding significance for the translation of clinical trials.

## Author contributions

All authors reviewed the literature and contributed to the preparation of this manuscript.

## References

[1] Heusch G. Cardioprotection research must leave its comfort zone. *Eur Heart J.* (2018) 39:3393–5. 10.1093/eurheartj/ehy253 29722801

[2] Hausenloy DJ Yellon DM. Ischaemic conditioning and reperfusion injury. *Nat Rev Cardiol.* (2016) 13:193–209. 10.1038/nrcardio.2016.5 26843289

[3] Davidson SM Ferdinandy P Andreadou I Botker HE Heusch G Ibanez B Multitarget strategies to reduce myocardial ischemia/reperfusion injury: JACC review topic of the week. *J Am Coll Cardiol.* (2019) 73:89–99. 10.1016/j.jacc.2018.09.086 30621955

[4] Heusch G Gersh BJ. The pathophysiology of acute myocardial infarction and strategies of protection beyond reperfusion: a continual challenge. *Eur Heart J.* (2017) 38:774–84. 10.1093/eurheartj/ehw224 27354052

[5] Bolli R. CAESAR’s legacy: a new era of rigor in preclinical studies of cardioprotection. *Basic Res Cardiol.* (2021) 116:33. 10.1007/s00395-021-00874-8 34018051 PMC8137617

[6] Heusch G. Critical issues for the translation of cardioprotection. *Circ Res.* (2017) 120:1477–86. 10.1161/CIRCRESAHA.117.310820 28450365

[7] Heusch G Rassaf T. Time to give up on cardioprotection? A critical appraisal of clinical studies on ischemic pre-, post-, and remote conditioning. *Circ Res.* (2016) 119:676–95. 10.1161/CIRCRESAHA.116.308736 27539973

[8] Jennings RB Sommers HM Smyth GA Flack HA Linn H. Myocardial necrosis induced by temporary occlusion of a coronary artery in the dog. *Arch Pathol.* (1960) 70:68–78. 14407094

[9] Bulluck H Hausenloy DJ. Ischaemic conditioning: are we there yet? *Heart.* (2015) 101:1067–77. 10.1136/heartjnl-2014-306531 25887783

[10] Heusch G. Molecular basis of cardioprotection: signal transduction in ischemic pre-, post-, and remote conditioning. *Circ Res.* (2015) 116:674–99. 10.1161/CIRCRESAHA.116.305348 25677517

[11] Shi W Vinten-Johansen J. Endogenous cardioprotection by ischaemic postconditioning and remote conditioning. *Cardiovasc Res.* (2012) 94:206–16. 10.1093/cvr/cvs088 22323534 PMC3331613

[12] Pilcher JM Young P Weatherall M Rahman I Bonser RS Beasley RW. A systematic review and meta-analysis of the cardioprotective effects of remote ischaemic preconditioning in open cardiac surgery. *J R Soc Med.* (2012) 105:436–45. 10.1258/jrsm.2012.120049 23104947 PMC3480853

[13] Rahman IA Mascaro JG Steeds RP Frenneaux MP Nightingale P Gosling P Remote ischemic preconditioning in human coronary artery bypass surgery: from promise to disappointment? *Circulation.* (2010) 122(11 Suppl.):S53–9. 10.1161/CIRCULATIONAHA.109.926667 20837926

[14] Kleinbongard P Skyschally A Heusch G. Cardioprotection by remote ischemic conditioning and its signal transduction. *Pflugers Arch.* (2017) 469:159–81. 10.1007/s00424-016-1922-6 27928644

[15] Lieder HR Tüller P Braczko F Zandi A Kamler M Thielmann M Bioassays of humoral cardioprotective factors released by remote ischemic conditioning in patients undergoing coronary artery bypass surgery. *J Cardiovasc Pharmacol Ther.* (2022) 27:10742484221097273. 10.1177/10742484221097273 35510644

[16] Lassen TR Just J Hjortbak MV Jespersen NR Stenz KT Gu T Cardioprotection by remote ischemic conditioning is transferable by plasma and mediated by extracellular vesicles. *Basic Res Cardiol.* (2021) 116:16. 10.1007/s00395-021-00856-w 33689033

[17] Donato M Buchholz B Rodríguez M Pérez V Inserte J García-Dorado D Role of the parasympathetic nervous system in cardioprotection by remote hindlimb ischaemic preconditioning. *Exp Physiol.* (2013) 98:425–34. 10.1113/expphysiol.2012.066217 22872660

[18] Mastitskaya S Marina N Gourine A Gilbey MP Spyer KM Teschemacher AG Cardioprotection evoked by remote ischaemic preconditioning is critically dependent on the activity of vagal pre-ganglionic neurones. *Cardiovasc Res.* (2012) 95:487–94. 10.1093/cvr/cvs212 22739118 PMC3422080

[19] Heusch G. Myocardial ischaemia-reperfusion injury and cardioprotection in perspective. *Nat Rev Cardiol.* (2020) 17:773–89. 10.1038/s41569-020-0403-y 32620851

[20] Basalay MV Yellon DM Davidson SM. Targeting myocardial ischaemic injury in the absence of reperfusion. *Basic Res Cardiol.* (2020) 115:63. 10.1007/s00395-020-00825-9 33057804 PMC7560937

[21] Hausenloy DJ Botker HE Ferdinandy P Heusch G Ng GA Redington A Cardiac innervation in acute myocardial ischaemia/reperfusion injury and cardioprotection. *Cardiovasc Res.* (2019) 115:1167–77. 10.1093/cvr/cvz053 30796814 PMC6529901

[22] Horton JL Virag J. Use of multifactorial treatments to address the challenge of translating experimental myocardial infarct reduction strategies. *Int J Mol Sci.* (2019) 20:1449. 10.3390/ijms20061449 30909376 PMC6471438

[23] Chen M Li X Yang H Tang J Zhou S. Hype or hope: vagus nerve stimulation against acute myocardial ischemia-reperfusion injury. *Trends Cardiovasc Med.* (2020) 30:481–8. 10.1016/j.tcm.2019.10.011 31740206

[24] Heusch G. Vagal cardioprotection in reperfused acute myocardial infarction. *JACC Cardiovasc Interv.* (2017) 10:1521–2. 10.1016/j.jcin.2017.05.063 28797428

[25] El Farissi M Keulards DCJ Zelis JM van Veer M Zimmermann FM Pijls NHJ Hypothermia for reduction of myocardial reperfusion injury in acute myocardial infarction: closing the translational gap. *Circ Cardiovasc Interv.* (2021) 14:e010326. 10.1161/CIRCINTERVENTIONS.120.010326 34266310

[26] Zhao S Qian J Wang J Gong P Yang Z Cahoon J Effects of oxygen concentrations on postresuscitation myocardial oxidative stress and myocardial function in a rat model of cardiopulmonary resuscitation. *Crit Care Med.* (2015) 43:e560–6. 10.1097/CCM.0000000000001297 26491859

[27] Bainey KR Armstrong PW. Clinical perspectives on reperfusion injury in acute myocardial infarction. *Am Heart J.* (2014) 167:637–45. 10.1016/j.ahj.2014.01.015 24766972

[28] Clemente-Moragon A Gomez M Villena-Gutierrez R Lalama DV Garcia-Prieto J Martinez F Metoprolol exerts a non-class effect against ischaemia-reperfusion injury by abrogating exacerbated inflammation. *Eur Heart J.* (2020) 41:4425–40. 10.1093/eurheartj/ehaa733 33026079 PMC7752252

[29] Kleinbongard P. Cardioprotection by early metoprolol- attenuation of ischemic vs. reperfusion injury? *Basic Res Cardiol.* (2020) 115:54. 10.1007/s00395-020-0814-2 32748009 PMC7399676

[30] Lobo-Gonzalez M Galán-Arriola C Rossello X González-Del-Hoyo M Vilchez JP Higuero-Verdejo MI Metoprolol blunts the time-dependent progression of infarct size. *Basic Res Cardiol.* (2020) 115:55. 10.1007/s00395-020-0812-4 32748088 PMC7398954

[31] Emelyanova L Bai X Yan Y Bosnjak ZJ Kress D Warner C Biphasic effect of metformin on human cardiac energetics. *Transl Res.* (2021) 229:5–23. 10.1016/j.trsl.2020.10.002 33045408 PMC10655614

[32] Griffiths K Lee JJ Frenneaux MP Feelisch M Madhani M. Nitrite and myocardial ischaemia reperfusion injury. Where are we now? *Pharmacol Ther.* (2021) 223:107819. 10.1016/j.pharmthera.2021.107819 33600852

[33] Rossello X Yellon DM. The RISK pathway and beyond. *Basic Res Cardiol.* (2018) 113:2. 10.1007/s00395-017-0662-x 29143177 PMC5688212

[34] Lu LQ Tian J Luo XJ Peng J. Targeting the pathways of regulated necrosis: a potential strategy for alleviation of cardio-cerebrovascular injury. *Cell Mol Life Sci.* (2021) 78:63–78. 10.1007/s00018-020-03587-8 32596778 PMC11072340

[35] Bøtker HE Cabrera-Fuentes HA Ruiz-Meana M Heusch G Ovize M. Translational issues for mitoprotective agents as adjunct to reperfusion therapy in patients with ST-segment elevation myocardial infarction. *J Cell Mol Med.* (2020) 24:2717–29. 10.1111/jcmm.14953 31967733 PMC7077531

[36] Shi Y Wang S Wu J Jin X You J. Pharmaceutical strategies for endoplasmic reticulum-targeting and their prospects of application. *J Control Release.* (2021) 329:337–52. 10.1016/j.jconrel.2020.11.054 33290795

[37] Ramachandra CJA Hernandez-Resendiz S Crespo-Avilan GE Lin YH Hausenloy DJ. Mitochondria in acute myocardial infarction and cardioprotection. *EBioMedicine.* (2020) 57:102884. 10.1016/j.ebiom.2020.102884 32653860 PMC7355051

[38] Sun K Li YY Jin J. A double-edged sword of immuno-microenvironment in cardiac homeostasis and injury repair. *Signal Transduct Target Ther.* (2021) 6:79. 10.1038/s41392-020-00455-6 33612829 PMC7897720

[39] Martins-Marques T Hausenloy DJ Sluijter JPG Leybaert L Girao H. Intercellular communication in the heart: therapeutic opportunities for cardiac ischemia. *Trends Mol Med.* (2021) 27:248–62. 10.1016/j.molmed.2020.10.002 33139169

[40] Kleinbongard P Andreadou I Vilahur G. The platelet paradox of injury versus protection in myocardial infarction-has it been overlooked? *Basic Res Cardiol.* (2021) 116:37. 10.1007/s00395-021-00876-6 34037862 PMC8150149

[41] Hausenloy DJ Chilian W Crea F Davidson SM Ferdinandy P Garcia-Dorado D The coronary circulation in acute myocardial ischaemia/reperfusion injury: a target for cardioprotection. *Cardiovasc Res.* (2019) 115:1143–55. 10.1093/cvr/cvy286 30428011 PMC6529918

[42] Davidson SM Andreadou I Barile L Birnbaum Y Cabrera-Fuentes HA Cohen MV Circulating blood cells and extracellular vesicles in acute cardioprotection. *Cardiovasc Res.* (2019) 115:1156–66. 10.1093/cvr/cvy314 30590395 PMC6529916

[43] Raphael Lieder H Tsoumani M Andreadou I Schrör K Heusch G Kleinbongard P. Platelet-mediated transfer of cardioprotection by remote ischemic conditioning and its abrogation by aspirin but not by Ticagrelor. *Cardiovasc Drugs Ther.* (2022). 10.1007/s10557-022-07345-9 [Epub ahead of print]. PMC10517043 35595877

[44] Zhou Z Mahdi A Tratsiakovich Y Zahoran S Kovamees O Nordin F erythrocytes from patients with type 2 diabetes induce endothelial dysfunction via arginase I. *J Am Coll Cardiol.* (2018) 72:769–80. 10.1016/j.jacc.2018.05.052 30092954

[45] Niccoli G Montone RA Ibanez B Thiele H Crea F Heusch G Optimized treatment of ST-elevation myocardial infarction. *Circ Res.* (2019) 125:245–58. 10.1161/CIRCRESAHA.119.315344 31268854

[46] Heusch G. Coronary microvascular obstruction: the new frontier in cardioprotection. *Basic Res Cardiol.* (2019) 114:45. 10.1007/s00395-019-0756-8 31617010

[47] Heusch G. The coronary circulation as a target of cardioprotection. *Circ Res.* (2016) 118:1643–58. 10.1161/CIRCRESAHA.116.308640 27174955

[48] Kleinbongard P Heusch G. A fresh look at coronary microembolization. *Nat Rev Cardiol.* (2022) 19:265–80. 10.1038/s41569-021-00632-2 34785770 PMC8593642

[49] O’Farrell FM Mastitskaya S Hammond-Haley M Freitas F Wah WR Attwell D. Capillary pericytes mediate coronary no-reflow after myocardial ischaemia. *Elife.* (2017) 6:e29280. 10.7554/eLife.29280 29120327 PMC5705208

[50] Bhatt DL Lopes RD Harrington RA. Diagnosis and treatment of acute coronary syndromes: a review. *JAMA.* (2022) 327:662–75. 10.1001/jama.2022.0358 35166796

[51] Jennings RB. Historical perspective on the pathology of myocardial ischemia/reperfusion injury. *Circ Res.* (2013) 113:428–38. 10.1161/CIRCRESAHA.113.300987 23908330

[52] Liu T Howarth AG Chen Y Nair AR Yang HJ Ren D Intramyocardial hemorrhage and the “wave front” of reperfusion injury compromising myocardial salvage. *J Am Coll Cardiol.* (2022) 79:35–48. 10.1016/j.jacc.2021.10.034 34991787 PMC13016961

[53] Jonassen AK Sack MN Mjos OD Yellon DM. Myocardial protection by insulin at reperfusion requires early administration and is mediated via Akt and p70s6 kinase cell-survival signaling. *Circ Res.* (2001) 89:1191–8. 10.1161/hh2401.101385 11739285

[54] Murugiah K Gupta A Krumholz HM. Time to reperfusion in ST-Segment elevation acute myocardial infarction: when does the clock start? *Circ Cardiovasc Interv.* (2021) 14:e010459. 10.1161/CIRCINTERVENTIONS.121.010459 33441005

[55] Cho KH Han X Ahn JH Hyun DY Kim MC Sim DS Long-Term outcomes of patients with late presentation of ST-segment elevation myocardial infarction. *J Am Coll Cardiol.* (2021) 77:1859–70. 10.1016/j.jacc.2021.02.041 33858622

[56] Braunwald E Kloner RA. Myocardial reperfusion: a double-edged sword? *J Clin Invest.* (1985) 76:1713–9. 10.1172/JCI112160 4056048 PMC424191

[57] Heusch G. Myocardial stunning and hibernation revisited. *Nat Rev Cardiol.* (2021) 18:522–36. 10.1038/s41569-021-00506-7 33531698

[58] Hausenloy DJ Ntsekhe M Yellon DM. A future for remote ischaemic conditioning in high-risk patients. *Basic Res Cardiol.* (2020) 115:35. 10.1007/s00395-020-0794-2 32335728

[59] Roth S Torregroza C Huhn R Hollmann MW Preckel B. Perioperative cardioprotection: clinical implications. *Anesthes Analg.* (2020) 131:1751–64. 10.1213/ANE.0000000000005234 33186162

[60] Heusch G Gersh BJ. Is cardioprotection salvageable? *Circulation.* (2020) 141:415–7. 10.1161/CIRCULATIONAHA.119.044176 32078426

[61] Yuan Y Liang B Liu XL Liu WJ Huang BH Yang SB Targeting NAD+: is it a common strategy to delay heart aging? *Cell Death Discov.* (2022) 8:230. 10.1038/s41420-022-01031-3 35474295 PMC9042931

[62] Wang R Wang M He S Sun G Sun X. Targeting calcium homeostasis in myocardial ischemia/reperfusion injury: an overview of regulatory mechanisms and therapeutic reagents. *Front Pharmacol.* (2020) 11:872. 10.3389/fphar.2020.00872 32581817 PMC7296066

[63] Ibáñez B Heusch G Ovize M Van de Werf F. Evolving therapies for myocardial ischemia/reperfusion injury. *J Am Coll Cardiol.* (2015) 65:1454–71. 10.1016/j.jacc.2015.02.032 25857912

[64] Wang J Zhou H. Mitochondrial quality control mechanisms as molecular targets in cardiac ischemia-reperfusion injury. *Acta Pharm Sin B.* (2020) 10:1866–79. 10.1016/j.apsb.2020.03.004 33163341 PMC7606115

[65] Li W Shi G. How Ca(V)1.2-bound verapamil blocks Ca(2+) influx into cardiomyocyte: atomic level views. *Pharmacol Res.* (2019) 139:153–7. 10.1016/j.phrs.2018.11.017 30447294

[66] Zhu H Zhou H. Novel insight into the role of endoplasmic reticulum stress in the pathogenesis of myocardial ischemia-reperfusion injury. *Oxid Med Cell Longev* (2021) 2021:5529810. 10.1155/2021/5529810 33854692 PMC8019635

[67] Wang K Li Y Qiang T Chen J Wang X. Role of epigenetic regulation in myocardial ischemia/reperfusion injury. *Pharmacol Res.* (2021) 170:105743. 10.1016/j.phrs.2021.105743 34182132

[68] Algoet M Janssens S Himmelreich U Gsell W Pusovnik M Van den Eynde J Myocardial ischemia-reperfusion injury and the influence of inflammation. *Trends Cardiovasc Med.* (2022) 22:S1050–738. 10.1016/j.tcm.2022.02.005 35181472

[69] Mishra PK Adameova A Hill JA Baines CP Kang PM Downey JM Guidelines for evaluating myocardial cell death. *Am J Physiol Heart Circ Physiol.* (2019) 317:H891–922. 10.1152/ajpheart.00259.2019 31418596 PMC6879915

[70] Frangogiannis NG. Pathophysiology of myocardial infarction. *Compr Physiol.* (2015) 5:1841–75. 10.1002/cphy.c150006 26426469

[71] Hausenloy DJ Yellon DM. Myocardial ischemia-reperfusion injury: a neglected therapeutic target. *J Clin Invest.* (2013) 123:92–100. 10.1172/JCI62874 23281415 PMC3533275

[72] Gottlieb RA. Cell death pathways in acute ischemia/reperfusion injury. *J Cardiovasc Pharmacol Ther.* (2011) 16:233–8. 10.1177/1074248411409581 21821521 PMC3337030

[73] Del Re DP Amgalan D Linkermann A Liu Q Kitsis RN. Fundamental mechanisms of regulated cell death and implications for heart disease. *Physiol Rev.* (2019) 99:1765–817. 10.1152/physrev.00022.2018 31364924 PMC6890986

[74] Taqueti VR Di Carli MF. Coronary microvascular disease pathogenic mechanisms and therapeutic options: JACC state-of-the-art review. *J Am Coll Cardiol.* (2018) 72:2625–41. 10.1016/j.jacc.2018.09.042 30466521 PMC6296779

[75] Kloner RA King KS Harrington MG. No-reflow phenomenon in the heart and brain. *Am J Physiol Heart Circ Physiol.* (2018) 315:H550–62. 10.1152/ajpheart.00183.2018 29882685

[76] Konijnenberg LSF Damman P Duncker DJ Kloner RA Nijveldt R van Geuns RM Pathophysiology and diagnosis of coronary microvascular dysfunction in ST-elevation myocardial infarction. *Cardiovasc Res.* (2020) 116:787–805. 10.1093/cvr/cvz301 31710673 PMC7061278

[77] Francone M Bucciarelli-Ducci C Carbone I Canali E Scardala R Calabrese FA Impact of primary coronary angioplasty delay on myocardial salvage, infarct size, and microvascular damage in patients with ST-segment elevation myocardial infarction: insight from cardiovascular magnetic resonance. *J Am Coll Cardiol.* (2009) 54:2145–53. 10.1016/j.jacc.2009.08.024 19942086

[78] Penna C Comità S Tullio F Alloatti G Pagliaro P. Challenges facing the clinical translation of cardioprotection: 35 years after the discovery of ischemic preconditioning. *Vasc Pharmacol.* (2022) 144:106995. 10.1016/j.vph.2022.106995 35470102

[79] Kleinbongard P Bøtker HE Ovize M Hausenloy DJ Heusch G. Co-morbidities and co-medications as confounders of cardioprotection-Does it matter in the clinical setting? *Br J Pharmacol.* (2020) 177:5252–69. 10.1111/bph.14839 31430831 PMC7680006

[80] Ferdinandy P Hausenloy DJ Heusch G Baxter GF Schulz R. Interaction of risk factors, comorbidities, and comedications with ischemia/reperfusion injury and cardioprotection by preconditioning, postconditioning, and remote conditioning. *Pharmacol Rev.* (2014) 66:1142–74. 10.1124/pr.113.008300 25261534

[81] Lecour S Andreadou I Bøtker HE Davidson SM Heusch G Ruiz-Meana M Improving preclinical assessment of cardioprotective therapies (IMPACT) criteria: guidelines of the EU-CARDIOPROTECTION COST action. *Basic Res Cardiol.* (2021) 116:52. 10.1007/s00395-021-00893-5 34515837 PMC8437922

[82] Penna C Andreadou I Aragno M Beauloye C Bertrand L Lazou A Effect of hyperglycaemia and diabetes on acute myocardial ischaemia-reperfusion injury and cardioprotection by ischaemic conditioning protocols. *Br J Pharmacol.* (2020) 177:5312–35. 10.1111/bph.14993 31985828 PMC7680002

[83] Crisafulli A Pagliaro P Roberto S Cugusi L Mercuro G Lazou A Diabetic cardiomyopathy and ischemic heart disease: prevention and therapy by exercise and conditioning. *Int J Mol Sci.* (2020) 21:2896. 10.3390/ijms21082896 PMC7215312 32326182

[84] Lejay A Fang F John R Van JA Barr M Thaveau F Ischemia reperfusion injury, ischemic conditioning and diabetes mellitus. *J Mol Cell Cardiol.* (2016) 91:11–22. 10.1016/j.yjmcc.2015.12.020 26718721

[85] Torregroza C Gnaegy L Raupach A Stroethoff M Feige K Heinen A Influence of hyperglycemia and diabetes on cardioprotection by humoral factors released after remote ischemic preconditioning (RIPC). *Int J Mol Sci* (2021) 22:8880. 10.3390/ijms22168880 34445586 PMC8396298

[86] Dewanjee S Vallamkondu J Kalra RS John A Reddy PH Kandimalla R. Autophagy in the diabetic heart: a potential pharmacotherapeutic target in diabetic cardiomyopathy. *Ageing Res Rev.* (2021) 68:101338. 10.1016/j.arr.2021.101338 33838320

[87] Qiao SG Sun Y Sun B Wang A Qiu J Hong L Sevoflurane postconditioning protects against myocardial ischemia/reperfusion injury by restoring autophagic flux via an NO-dependent mechanism. *Acta Pharmacol Sin.* (2019) 40:35–45. 10.1038/s41401-018-0066-y 30002490 PMC6318323

[88] Gao S Wang R Dong S Wu J Perek B Xia Z Inactivation of TOPK Caused by Hyperglycemia Blocks Diabetic Heart Sensitivity to Sevoflurane Postconditioning by Impairing the PTEN/PI3K/Akt Signaling. *Oxid Med Cell Longev.* (2021) 2021:6657529. 10.1155/2021/6657529 33986917 PMC8093075

[89] Gedik N Thielmann M Kottenberg E Peters J Jakob H Heusch G No evidence for activated autophagy in left ventricular myocardium at early reperfusion with protection by remote ischemic preconditioning in patients undergoing coronary artery bypass grafting. *PLoS One.* (2014) 9:e96567. 10.1371/journal.pone.0096567 24797938 PMC4010496

[90] Mokhtari B Badalzadeh R. The potentials of distinct functions of autophagy to be targeted for attenuation of myocardial ischemia/reperfusion injury in preclinical studies: an up-to-date review. *J Physiol Biochem.* (2021) 77:377–404. 10.1007/s13105-021-00824-x 34173955

[91] Mokhtari B Abdoli-Shadbad M Alihemmati A Javadi A Badalzadeh R. Alpha-lipoic acid preconditioning plus ischemic postconditioning provides additional protection against myocardial reperfusion injury of diabetic rats: modulation of autophagy and mitochondrial function. *Mol Biol Rep.* (2022) 49:1773–82. 10.1007/s11033-021-06987-6 35098396

[92] Miki T Itoh T Sunaga D Miura T. Effects of diabetes on myocardial infarct size and cardioprotection by preconditioning and postconditioning. *Cardiovasc Diabetol.* (2012) 11:67. 10.1186/1475-2840-11-67 22694800 PMC3461466

[93] Ran J Xu H Li W. Cardioprotective effects of co-administration of thymoquinone and ischemic postconditioning in diabetic rats. *Iran J Basic Med Sci.* (2021) 24:892–9. 34712418 10.22038/ijbms.2021.47670.10981 PMC8528251

[94] Badalzadeh R Azimi A Alihemmati A Yousefi B. Chronic type-I diabetes could not impede the anti-inflammatory and anti-apoptotic effects of combined postconditioning with ischemia and cyclosporine A in myocardial reperfusion injury. *J Physiol Biochem.* (2017) 73:111–20. 10.1007/s13105-016-0530-4 27771871

[95] Zhang J Cai X Zhang Q Li X Li S Ma J Hydrogen sulfide restores sevoflurane postconditioning mediated cardioprotection in diabetic rats: role of SIRT1/Nrf2 signaling-modulated mitochondrial dysfunction and oxidative stress. *J Cell Physiol.* (2021) 236:5052–68. 10.1002/jcp.30214 33325044

[96] Lin J Wang T Li Y Wang M Li H Irwin MG N-Acetylcysteine restores sevoflurane postconditioning cardioprotection against myocardial ischemia-reperfusion injury in diabetic rats. *J Diabetes Res.* (2016) 2016:9213034. 10.1155/2016/9213034 26783539 PMC4691468

[97] Yang L Xie P Wu J Yu J Li X Ma H Deferoxamine Treatment combined with sevoflurane postconditioning attenuates myocardial ischemia-reperfusion injury by restoring HIF-1/BNIP3-mediated mitochondrial autophagy in GK rats. *Front Pharmacol.* (2020) 11:6. 10.3389/fphar.2020.00006 32140105 PMC7042377

[98] Xie P Yang L Talaiti A Wu JJ Yu J Yu T Deferoxamine-activated hypoxia-inducible factor-1 restores cardioprotective effects of sevoflurane postconditioning in diabetic rats. *Acta Physiol.* (2017) 221:98–114. 10.1111/apha.12874 28316125

[99] Zou R Nie C Pan S Wang B Hong X Xi S Co-administration of hydrogen and metformin exerts cardioprotective effects by inhibiting pyroptosis and fibrosis in diabetic cardiomyopathy. *Free Radic Biol Med.* (2022) 183:35–50. 10.1016/j.freeradbiomed.2022.03.010 35304269

[100] Zykov VA Tuchina TP Lebedev DA Krylova IB Babenko AY Kuleshova EV Effects of glucagon-like peptide 1 analogs in combination with insulin on myocardial infarct size in rats with type 2 diabetes mellitus. *World J Diabetes.* (2018) 9:149–56. 10.4239/wjd.v9.i9.149 30254724 PMC6153122

[101] Bayrami G Karimi P Agha-Hosseini F Feyzizadeh S Badalzadeh R. Effect of ischemic postconditioning on myocardial function and infarct size following reperfusion injury in diabetic rats pretreated with vildagliptin. *J Cardiovasc Pharmacol Ther.* (2018) 23:174–83. 10.1177/1074248417729881 28901167

[102] Alburquerque-Béjar JJ Barba I Inserte J Miró-Casas E Ruiz-Meana M Poncelas M Combination therapy with remote ischaemic conditioning and insulin or exenatide enhances infarct size limitation in pigs. *Cardiovasc Res.* (2015) 107:246–54. 10.1093/cvr/cvv171 26045476

[103] Jeddi S Gheibi S Afzali H Carlström M Kashfi K Ghasemi A. Hydrogen sulfide potentiates the protective effects of nitrite against myocardial ischemia-reperfusion injury in type 2 diabetic rats. *Nitric Oxide.* (2022) 124:15–23. 10.1016/j.niox.2022.04.004 35504499

[104] Matsumoto S Cho S Tosaka S Higashijima U Maekawa T Hara T Hyperglycemia raises the threshold of levosimendan- but not milrinone-induced postconditioning in rat hearts. *Cardiovasc Diabetol.* (2012) 11:4. 10.1186/1475-2840-11-4 22239823 PMC3269349

[105] Torregroza C Yueksel B Ruske R Stroethoff M Raupach A Heinen A Combination of cyclosporine A and levosimendan induces cardioprotection under acute hyperglycemia. *Int J Mol Sci.* (2021) 22:4517. 10.3390/ijms22094517 33926009 PMC8123582

[106] Baumgardt SL Paterson M Leucker TM Fang J Zhang DX Bosnjak ZJ Chronic co-administration of sepiapterin and l-citrulline ameliorates diabetic cardiomyopathy and myocardial ischemia/reperfusion injury in obese type 2 diabetic mice. *Circ Heart Fail.* (2016) 9:e002424. 10.1161/CIRCHEARTFAILURE.115.002424 26763290 PMC4714787

[107] Yu LM Dong X Xue XD Xu S Zhang X Xu YL Melatonin attenuates diabetic cardiomyopathy and reduces myocardial vulnerability to ischemia-reperfusion injury by improving mitochondrial quality control: role of SIRT6. *J Pineal Res.* (2021) 70:e12698. 10.1111/jpi.12698 33016468

[108] King N Al Shaama M Suleiman MS. Propofol improves recovery of the isolated working hypertrophic heart from ischaemia-reperfusion. *Pflugers Arch.* (2012) 464:513–22. 10.1007/s00424-012-1152-5 23001119

[109] Shao Q Casin KM Mackowski N Murphy E Steenbergen C Kohr MJ. Adenosine A1 receptor activation increases myocardial protein S-nitrosothiols and elicits protection from ischemia-reperfusion injury in male and female hearts. *PLoS One.* (2017) 12:e0177315. 10.1371/journal.pone.0177315 28493997 PMC5426678

[110] Penna C Tullio F Moro F Folino A Merlino A Pagliaro P. Effects of a protocol of ischemic postconditioning and/or captopril in hearts of normotensive and hypertensive rats. *Basic Res Cardiol.* (2010) 105:181–92. 10.1007/s00395-009-0075-6 20012872

[111] Penna C Tullio F Perrelli MG Moro F Abbadessa G Piccione F Ischemia/reperfusion injury is increased and cardioprotection by a postconditioning protocol is lost as cardiac hypertrophy develops in nandrolone treated rats. *Basic Res Cardiol.* (2011) 106:409–20. 10.1007/s00395-010-0143-y 21174210

[112] Jalowy A Schulz R Dörge H Behrends M Heusch G. Infarct size reduction by AT1-receptor blockade through a signal cascade of AT2-receptor activation, bradykinin and prostaglandins in pigs. *J Am Coll Cardiol.* (1998) 32:1787–96. 10.1016/S0735-1097(98)00441-0 9822110

[113] Ehring T Baumgart D Krajcar M Hümmelgen M Kompa S Heusch G. Attenuation of myocardial stunning by the ACE inhibitor ramiprilat through a signal cascade of bradykinin and prostaglandins but not nitric oxide. *Circulation.* (1994) 90:1368–85. 10.1161/01.CIR.90.3.1368 8087948

[114] Husková Z Kikerlová S Sadowski J Alánová P Sedláková L Papoušek F Increased endogenous activity of the renin-angiotensin system reduces infarct size in the rats with early angiotensin ii-dependent hypertension which survive the acute ischemia/reperfusion injury. *Front Pharmacol.* (2021) 12:679060. 10.3389/fphar.2021.679060 34122103 PMC8193500

[115] Ferdinandy P Schulz R Baxter GF. Interaction of cardiovascular risk factors with myocardial ischemia/reperfusion injury, preconditioning, and postconditioning. *Pharmacol Rev.* (2007) 59:418–58. 10.1124/pr.107.06002 18048761

[116] Lu X Bi YW Chen KB. Olmesartan restores the protective effect of remote ischemic perconditioning against myocardial ischemia/reperfusion injury in spontaneously hypertensive rats. *Clinics.* (2015) 70:500–7. 10.6061/clinics/2015(07)07 26222820 PMC4496757

[117] Polhemus DJ Gao J Scarborough AL Trivedi R McDonough KH Goodchild TT Radiofrequency Renal Denervation Protects the Ischemic Heart via Inhibition of GRK2 and Increased Nitric Oxide Signaling. *Circ Res.* (2016) 119:470–80. 10.1161/CIRCRESAHA.115.308278 27296507 PMC4959827

[118] Polhemus DJ Trivedi RK Gao J Li Z Scarborough AL Goodchild TT Renal sympathetic denervation protects the failing heart via inhibition of neprilysin activity in the kidney. *J Am Coll Cardiol.* (2017) 70:2139–53. 10.1016/j.jacc.2017.08.056 29050562

[119] Polhemus DJ Trivedi RK Sharp TE Li Z Goodchild TT Scarborough A Repeated cell transplantation and adjunct renal denervation in ischemic heart failure: exploring modalities for improving cell therapy efficacy. *Basic Res Cardiol.* (2019) 114:9. 10.1007/s00395-019-0718-1 30656501 PMC7121860

[120] Sun X Wei Z Li Y Wang J Hu J Yin Y Renal denervation restrains the inflammatory response in myocardial ischemia-reperfusion injury. *Basic Res Cardiol.* (2020) 115:15. 10.1007/s00395-020-0776-4 31932910

[121] Andreadou I Iliodromitis EK Lazou A Görbe A Giricz Z Schulz R Effect of hypercholesterolaemia on myocardial function, ischaemia-reperfusion injury and cardioprotection by preconditioning, postconditioning and remote conditioning. *Br J Pharmacol.* (2017) 174:1555–69. 10.1111/bph.13704 28060997 PMC5446572

[122] Osipov RM Bianchi C Feng J Clements RT Liu Y Robich MP Effect of hypercholesterolemia on myocardial necrosis and apoptosis in the setting of ischemia-reperfusion. *Circulation.* (2009) 120(11 Suppl.):S22–30. 10.1161/CIRCULATIONAHA.108.842724 19752371 PMC2752874

[123] Roth S Torregroza C Feige K Preckel B Hollmann MW Weber NC Pharmacological conditioning of the heart: an update on experimental developments and clinical implications. *Int J Mol Sci.* (2021) 22:2519. 10.3390/ijms22052519 33802308 PMC7959135

[124] Ma LL Kong FJ Guo JJ Zhu JB Shi HT Li Y Hypercholesterolemia abrogates remote ischemic preconditioning-induced cardioprotection: role of reperfusion injury salvage kinase signals. *Shock.* (2017) 47:363–9. 10.1097/SHK.0000000000000737 27559699

[125] Görbe A Varga ZV Kupai K Bencsik P Kocsis GF Csont T Cholesterol diet leads to attenuation of ischemic preconditioning-induced cardiac protection: the role of connexin 43. *Am J Physiol Heart Circ Physiol.* (2011) 300:H1907–13. 10.1152/ajpheart.01242.2010 21398600

[126] Tang XL Stein AB Shirk G Bolli R. Hypercholesterolemia blunts NO donor-induced late preconditioning against myocardial infarction in conscious rabbits. *Basic Res Cardiol.* (2004) 99:395–403. 10.1007/s00395-004-0485-4 15372283 PMC3713468

[127] Yadav HN Singh M Sharma PL. Pharmacological inhibition of GSK-3β produces late phase of cardioprotection in hyperlipidemic rat: possible involvement of HSP 72. *Mol Cell Biochem.* (2012) 369:227–33. 10.1007/s11010-012-1386-8 22810500

[128] Zhao H Wang Y Wu Y Li X Yang G Ma X Hyperlipidemia does not prevent the cardioprotection by postconditioning against myocardial ischemia/reperfusion injury and the involvement of hypoxia inducible factor-1alpha upregulation. *Acta Biochim Biophys Sin.* (2009) 41:745–53. 10.1093/abbs/gmp063 19727523

[129] Csonka C Kupai K Bencsik P Görbe A Pálóczi J Zvara A Cholesterol-enriched diet inhibits cardioprotection by ATP-sensitive K+ channel activators cromakalim and diazoxide. *Am J Physiol Heart Circ Physiol.* (2014) 306:H405–13. 10.1152/ajpheart.00257.2013 24285110

[130] Huhn R Heinen A Hollmann MW Schlack W Preckel B Weber NC. Cyclosporine A administered during reperfusion fails to restore cardioprotection in prediabetic Zucker obese rats in vivo. *Nutr Metab Cardiovasc Dis.* (2010) 20:706–12. 10.1016/j.numecd.2009.06.010 19819119

[131] Wu N Li WN Shu WQ Lv Y Jia DL. Blocking the mitochondrial permeability transition pore with cyclosporine-A can restore cardioprotection of ischemic postconditioning in hypercholesterolemic rat heart. *Eur Rev Med Pharmacol Sci.* (2015) 19:446–54. 25720717

[132] Gömöri K Szabados T Kenyeres É Pipis J Földesi I Siska A Cardioprotective effect of novel matrix metalloproteinase inhibitors. *Int J Mol Sci.* (2020) 21:6990. 10.3390/ijms21196990 PMC7582346 32977437

[133] Bell RM Kunuthur SP Hendry C Bruce-Hickman D Davidson S Yellon DM. Matrix metalloproteinase inhibition protects CyPD knockout mice independently of RISK/mPTP signalling: a parallel pathway to protection. *Basic Res Cardiol.* (2013) 108:331. 10.1007/s00395-013-0331-7 23361433

[134] Hong J Ge HW Liu JQ Sun RH Kong FJ. Pharmacological inhibition of PTEN restores remote ischemic postconditioning cardioprotection in hypercholesterolemic mice: potential role of PTEN/AKT/GSK3β SIGNALS. *Shock.* (2019) 52:522–31. 10.1097/SHK.0000000000001296 30499878

[135] Nikolaou PE Boengler K Efentakis P Vouvogiannopoulou K Zoga A Gaboriaud-Kolar N Investigating and re-evaluating the role of glycogen synthase kinase 3 beta kinase as a molecular target for cardioprotection by using novel pharmacological inhibitors. *Cardiovasc Res.* (2019) 115:1228–43. 10.1093/cvr/cvz061 30843027

[136] Andreadou I Farmakis D Prokovas E Sigala F Zoga A Spyridaki K Short-term statin administration in hypercholesterolaemic rabbits resistant to postconditioning: effects on infarct size, endothelial nitric oxide synthase, and nitro-oxidative stress. *Cardiovasc Res.* (2012) 94:501–9. 10.1093/cvr/cvs121 22411971

[137] Boengler K Schulz R Heusch G. Loss of cardioprotection with ageing. *Cardiovasc Res.* (2009) 83:247–61. 10.1093/cvr/cvp033 19176601

[138] van den Munckhof I Riksen N Seeger JP Schreuder TH Borm GF Eijsvogels TM Aging attenuates the protective effect of ischemic preconditioning against endothelial ischemia-reperfusion injury in humans. *Am J Physiol Heart Circ Physiol.* (2013) 304:H1727–32. 10.1152/ajpheart.00054.2013 23604707

[139] Heusch G Boengler K Schulz R. Cardioprotection: nitric oxide, protein kinases, and mitochondria. *Circulation.* (2008) 118:1915–9. 10.1161/CIRCULATIONAHA.108.805242 18981312

[140] Chen J Gao J Sun W Li L Wang Y Bai S Involvement of exogenous H2S in recovery of cardioprotection from ischemic post-conditioning via increase of autophagy in the aged hearts. *Int J Cardiol.* (2016) 220:681–92. 10.1016/j.ijcard.2016.06.200 27393850

[141] Rajabi M Vafaee MS Hosseini L Badalzadeh R. Pretreatment with nicotinamide mononucleotide increases the effect of ischaemic postconditioning on cardioprotection and mitochondrial function following ex vivo myocardial reperfusion injury in aged rats. *Clin Exp Pharmacol Physiol.* (2022) 49:474–82. 10.1111/1440-1681.13616 34854121

[142] Abete P Testa G Galizia G Mazzella F Della Morte D de Santis D Tandem action of exercise training and food restriction completely preserves ischemic preconditioning in the aging heart. *Exp Gerontol.* (2005) 40:43–50. 10.1016/j.exger.2004.10.005 15664731

[143] Ruiz-Meana M Bou-Teen D Ferdinandy P Gyongyosi M Pesce M Perrino C Cardiomyocyte ageing and cardioprotection: consensus document from the ESC working groups cell biology of the heart and myocardial function. *Cardiovasc Res.* (2020) 116:1835–49. 10.1093/cvr/cvaa132 32384145

[144] Hosseini L Vafaee MS Badalzadeh R. Melatonin and nicotinamide mononucleotide attenuate myocardial ischemia/reperfusion injury via modulation of mitochondrial function and hemodynamic parameters in aged rats. *J Cardiovasc Pharmacol Ther.* (2020) 25:240–50. 10.1177/1074248419882002 31645107

[145] Petrosillo G Moro N Paradies V Ruggiero FM Paradies G. Increased susceptibility to Ca(2+)-induced permeability transition and to cytochrome c release in rat heart mitochondria with aging: effect of melatonin. *J Pineal Res.* (2010) 48:340–6. 10.1111/j.1600-079X.2010.00758.x 20345745

[146] Li C Mu N Gu C Liu M Yang Z Yin Y Metformin mediates cardioprotection against aging-induced ischemic necroptosis. *Aging cell.* (2020) 19:e13096. 10.1111/acel.13096 31944526 PMC6996959

[147] Nakao A Kaczorowski DJ Wang Y Cardinal JS Buchholz BM Sugimoto R Amelioration of rat cardiac cold ischemia/reperfusion injury with inhaled hydrogen or carbon monoxide, or both. *J Heart Lung Transplant.* (2010) 29:544–53. 10.1016/j.healun.2009.10.011 20036162

[148] Díaz-Vesga MC Zúñiga-Cuevas Ú Ramírez-Reyes A Herrera-Zelada N Palomo I Bravo-Sagua R Potential therapies to protect the aging heart against ischemia/reperfusion injury. *Front Cardiovasc Med.* (2021) 8:770421. 10.3389/fcvm.2021.770421 34869687 PMC8639870

[149] Rossello X Yellon DM. Cardioprotection: the disconnect between bench and bedside. *Circulation.* (2016) 134:574–5. 10.1161/CIRCULATIONAHA.116.022829 27550967

[150] Hausenloy DJ Botker HE Engstrom T Erlinge D Heusch G Ibanez B Targeting reperfusion injury in patients with ST-segment elevation myocardial infarction: trials and tribulations. *Eur Heart J.* (2017) 38:935–41. 10.1093/eurheartj/ehw145 27118196 PMC5381598

[151] Bøtker HE Hausenloy D Andreadou I Antonucci S Boengler K Davidson SM Practical guidelines for rigor and reproducibility in preclinical and clinical studies on cardioprotection. *Basic Res Cardiol.* (2018) 113:39. 10.1007/s00395-018-0696-8 30120595 PMC6105267

[152] Ibanez B Aletras AH Arai AE Arheden H Bax J Berry C Cardiac MRI endpoints in myocardial infarction experimental and clinical trials: JACC scientific expert panel. *J Am Coll Cardiol.* (2019) 74:238–56. 10.1016/j.jacc.2019.05.024 31296297 PMC7363031

[153] García Del Blanco B Otaegui I Rodríguez-Palomares JF Bayés-Genis A Fernández-Nofrerías E Vilalta Del Olmo V Effect of COMBinAtion therapy with remote ischemic conditioning and exenatide on the Myocardial Infarct size: a two-by-two factorial randomized trial (COMBAT-MI). *Basic Res Cardiol.* (2021) 116:4. 10.1007/s00395-021-00842-2 33495853

[154] Lindsey ML Bolli R Canty JM Jr Du XJ Frangogiannis NG Frantz S Guidelines for experimental models of myocardial ischemia and infarction. *Am J Physiol Heart Circ Physiol.* (2018) 314:H812–38. 10.1152/ajpheart.00335.2017 29351451 PMC5966768

[155] Lindsey ML Brunt KR Kirk JA Kleinbongard P Calvert JW de Castro Brás LE Guidelines for in vivo mouse models of myocardial infarction. *Am J Physiol Heart Circ Physiol.* (2021) 321:H1056–73. 10.1152/ajpheart.00459.2021 34623181 PMC8834230

[156] Acun A Nguyen TD Zorlutuna P. In vitro aged, hiPSC-origin engineered heart tissue models with age-dependent functional deterioration to study myocardial infarction. *Acta biomater.* (2019) 94:372–91. 10.1016/j.actbio.2019.05.064 31146032 PMC6779061

[157] Perbellini F Thum T. Living myocardial slices: a novel multicellular model for cardiac translational research. *Eur Heart J.* (2020) 41:2405–8. 10.1093/eurheartj/ehz779 31711161 PMC7327529

[158] Watson SA Duff J Bardi I Zabielska M Atanur SS Jabbour RJ Biomimetic electromechanical stimulation to maintain adult myocardial slices in vitro. *Nat Commun.* (2019) 10:2168. 10.1038/s41467-019-10175-3 31092830 PMC6520377

[159] Kleinbongard P Gedik N Kirca M Stoian L Frey U Zandi A Mitochondrial and contractile function of human right atrial tissue in response to remote ischemic conditioning. *J Am Heart Assoc.* (2018) 7:e009540. 10.1161/JAHA.118.009540 30371229 PMC6201459

[160] Ale-Agha N Jakobs P Goy C Zurek M Rosen J Dyballa-Rukes N Mitochondrial telomerase reverse transcriptase protects from myocardial ischemia/reperfusion injury by improving complex i composition and function. *Circulation.* (2021) 144:1876–90. 10.1161/CIRCULATIONAHA.120.051923 34672678

[161] Feng HZ Jin JP. A protocol to study ex vivo mouse working heart at human-like heart rate. *J Mol Cell Cardiol.* (2018) 114:175–84. 10.1016/j.yjmcc.2017.11.011 29155072 PMC5801060

[162] He Z Davidson SM Yellon DM. The importance of clinically relevant background therapy in cardioprotective studies. *Basic Res Cardiol.* (2020) 115:69. 10.1007/s00395-020-00830-y 33188438 PMC7666584

[163] Kleinbongard P Neuhäuser M Thielmann M Kottenberg E Peters J Jakob H Confounders of cardioprotection by remote ischemic preconditioning in patients undergoing coronary artery bypass grafting. *Cardiology.* (2016) 133:128–33. 10.1159/000441216 26536214

[164] Goodman SN Fanelli D Ioannidis JP. What does research reproducibility mean? *Sci Transl Med.* (2016) 8:341s12. 10.1126/scitranslmed.aaf5027 27252173

[165] Baxter GF Hale SL Miki T Kloner RA Cohen MV Downey JM Adenosine A1 agonist at reperfusion trial (AART): results of a three-center, blinded, randomized, controlled experimental infarct study. *Cardiovasc Drugs Ther.* (2000) 14:607–14. 10.1023/A:1007850527878 11300361

[166] Kim B Trounson A. Design preclinical studies for reproducibility. *Nat Biomed Eng.* (2018) 2:789–90. 10.1038/s41551-018-0322-y 31015618

[167] Lefer DJ Bolli R. Development of an NIH consortium for preclinicAl AssESsment of CARdioprotective therapies (CAESAR): a paradigm shift in studies of infarct size limitation. *J Cardiovasc Pharmacol Ther.* (2011) 16:332–9. 10.1177/1074248411414155 21821536

[168] Jones SP Tang XL Guo Y Steenbergen C Lefer DJ Kukreja RC The NHLBI-sponsored Consortium for preclinicAl assESsment of cARdioprotective therapies (CAESAR): a new paradigm for rigorous, accurate, and reproducible evaluation of putative infarct-sparing interventions in mice, rabbits, and pigs. *Circ Res.* (2015) 116:572–86. 10.1161/CIRCRESAHA.116.305462 25499773 PMC4329104

[169] Rossello X Rodriguez-Sinovas A Vilahur G Crisóstomo V Jorge I Zaragoza C CIBER-CLAP (CIBERCV Cardioprotection Large Animal Platform): a multicenter preclinical network for testing reproducibility in cardiovascular interventions. *Sci Rep.* (2019) 9:20290. 10.1038/s41598-019-56613-6 31889088 PMC6937304

